# Current status of use of high throughput nucleotide sequencing in rheumatology

**DOI:** 10.1136/rmdopen-2020-001324

**Published:** 2021-01-06

**Authors:** Sebastian Boegel, John C Castle, Andreas Schwarting

**Affiliations:** 1 Department of Internal Medicine, University Center of Autoimmunity, University Medical Center Mainz, Mainz, Germany; 2 Monte Rosa Therapeutics, Basel, Switzerland; 3 Division of Rheumatology and Clinical Immunology, University Hospital Mainz, Mainz, Germany; 4 Acura Rheumatology Center Rhineland Palatinate, Bad Kreuznach, Germany

**Keywords:** lupus erythematosus, systemic, arthritis, rheumatoid, osteoarthritis, dermatomyositis, familial mediterranean fever

## Abstract

**Objective:**

Here, we assess the usage of high throughput sequencing (HTS) in rheumatic research and the availability of public HTS data of rheumatic samples.

**Methods:**

We performed a semiautomated literature review on PubMed, consisting of an R-script and manual curation as well as a manual search on the Sequence Read Archive for public available HTS data.

**Results:**

Of the 699 identified articles, rheumatoid arthritis (n=182 publications, 26%), systemic lupus erythematous (n=161, 23%) and osteoarthritis (n=152, 22%) are among the rheumatic diseases with the most reported use of HTS assays. The most represented assay is RNA-Seq (n=457, 65%) for the identification of biomarkers in blood or synovial tissue. We also find, that the quality of accompanying clinical characterisation of the sequenced patients differs dramatically and we propose a minimal set of clinical data necessary to accompany rheumatological-relevant HTS data.

**Conclusion:**

HTS allows the analysis of a broad spectrum of molecular features in many samples at the same time. It offers enormous potential in novel personalised diagnosis and treatment strategies for patients with rheumatic diseases. Being established in cancer research and in the field of Mendelian diseases, rheumatic diseases are about to become the third disease domain for HTS, especially the RNA-Seq assay. However, we need to start a discussion about reporting of clinical characterisation accompany rheumatological-relevant HTS data to make clinical meaningful use of this data.

**Key messages**

**What is already known about this subject?**

- High throughput sequencing (HTS) has enormous potential in rheumatic research as it offers a broad spectrum of molecular analysis.
- While widely adopted in cancer research, the usage of the various HTS assays in rheumatological research has not been quantified.

**What does the study add?**

- HTS is being adapted in rheumatological research, with rheumatoid arthritis and systemic lupus erythematous as the major indications and RNA-Seq as the most represented HTS assay.
- The quality of accompanying clinical characterisation of the sequenced patients differs dramatically.

**How might this impact on clinical practice or future developments?**

- Rheumatic diseases are about to become the third disease domain for HTS, however, here we start a discussion of reporting sequencing data by proposing a minimal set of clinical data necessary to accompany rheumatological-relevant HTS data.

## Introduction

The aim of ‘precision medicine’ is the development of novel diagnosis, prevention and treatment strategies by taking into account the individuality of a patient 1 including the individual molecular profile.^2^ The development of high throughput sequencing (HTS) platforms, collectively still called ‘next-generation sequencing’ (NGS), allows a comprehensive and multimodal molecular profile of a patient. In particular, gene expression analysis using whole- transcriptome sequencing (RNA-Seq) has become state-of-the-art 3 as it has been demonstrated to be more accurate, sensitive, as well as to have a broader dynamic range than DNA microarrays allowing the detection of more differentially expressed genes with higher fold change.^4^ In addition, this assay provides both: abundance of transcripts and sequence information at base-pair resolution, thus allowing a broad spectrum of analyses beyond gene and transcript expression, enabling the detection of a wide variety of molecular features, such as alternative splicing events, RNA editing events, complementarity determining region 3 of T cell receptors (TCRs), B cell receptors (BCR), human leucocyte antigen (HLA) types.^5^ In addition, HTS of exons, such as whole exome sequencing (WES) or targeted sequencing (gene panels), allows the rapid detection of DNA-encoded variants, such tumour cell mutations, and is a key technology enabling the development of mutanome-based cancer immunotherapies.^6^ Not only has the adoption of HTS has been rapid in oncology, but clinical and research laboratories worldwide have made primary sequencing data available in the Sequence Read Archive (SRA, http://www.ncbi.nlm.nih.gov/sra),^7^ one of the largest data repositories with 7.5 PB of open-access HTS data.^8^ The repository comprises data from over 340 000 samples 9 and thus provides a rich and valuable source for reanalysis of existing datasets with bioinformatic software 5 to identify novel and clinical translatable findings.

Moreover, non-invasive and minimally invasive profiling platforms, including ‘liquid biopsies’, allow one to obtain information about a disease state or response to treatment using, for example, blood from patients, followed by HTS profiling and subsequent bioinformatic analysis. While this concept is already implemented in oncology,^10^ it is less mature in rheumatology. We argue here that HTS offers enormous potential to pave the way to personalised therapy 11 for patients with rheumatic diseases, particularly due to its extreme molecular and phenotypic heterogeneity 12.

Very recently in this journal, Kedra *et al* 13 reviewed the current use of big data and artificial intelligence in rheumatic diseases. Here, we focus on HTS profiling as a big data producer 14 and review both the literature using HTS and public HTS datasets in rheumatological diseases to quantify the adoption of this technology in rheumatology. In addition, we propose a minimal set of clinical data necessary to accompany rheumatological-relevant HTS data.

## Methods

### Systematic literature review

The literature review was implemented in *R* (V.3.6.1,^15^) using the package easyPubMed (V.2.13,^16^) and consists of 2 steps. First an automated PubMed search was carried on 15 August 2020 out using the query string:

“(methylomics OR epigenomics OR NGS OR \“next generation sequencing\” OR RNA-Seq OR \“mRNA sequencing\” OR \“RNA sequencing\” OR \“RNA-sequencing\” OR \“transcriptome sequencing\” OR \“whole exome sequencing\” OR \“whole-exome sequencing\” OR \“high throughput sequencing\” OR \“high-throughput sequencing\” OR \“DNA sequencing\” OR \“RNA sequencing\” OR \“RNA-sequencing\” OR \“DNA-sequencing\” OR WXS OR WGS OR \“whole-genome sequencing\” OR \“whole genome sequencing\“) AND (rheumatology OR \“rheumatologic disease\” OR \“rheumatologic disease\”))".

This search resulted in 1097 entries. The keywords of each returning dataset were intersected with official disease names extracted from International Statistical Classification of Diseases and Related Health Problems (ICD)-11^17^ in order to filter out keywords that are not disease names. The remaining 253 keywords were then manually inspected to find rheumatic diseases. This approach identified the following diseases: autoinflammatory syndrome, dermatomyositis, enthesitis, familial mediterranean fever (FMF), granulomatosis with polyangiitis (GPA), juvenile idiopathic arthritis (JIA), myositis, osteoarthritis (OA), polymyositis, psoriatic arthritis (PsA), rheumatoid arthritis (RA), sacroiliitis, sjögren’s syndrome, spondyloarthritis (SpA), synovitis, systemic lupus erythematosus (SLE), systemic sclerosis vasculitis, uveitis, gout and polychondritis.

In a second step more specific PubMed search was carried out using the disease names identified in the first step:

“(methylomics OR epigenomics OR NGS OR \“next generation sequencing\” OR RNA-Seq OR \“mRNA sequencing\” OR \“RNA sequencing\” OR \“RNA-sequencing\” OR \“transcriptome sequencing\” OR \“whole exome sequencing\” OR \“whole-exome sequencing\” OR \“high throughput sequencing\” OR \“high-throughput sequencing\” OR WXS OR WGS OR \“whole-genome sequencing\” OR \“whole genome sequencing\“) AND (\“autoinflammatory syndrome\” OR dermatomyositis OR enthesitis OR \“familial mediterranean fever\” OR \“granulomatosis with polyangiitis\” OR \“juvenile idiopathic arthritis\” OR myositis OR osteoarthritis OR polymyositis OR \“psoriatic arthritis\” OR \“rheumatoid arthritis\” OR sacroiliitis OR \“sjögren syndrome\” OR \“sjögren’s syndrome\” OR spondyloarthritis OR synovitis OR \“systemic lupus erythematosus\” OR \“systemic sclerosis\” OR vasculitis OR uveitis OR gout OR polychondritis)".

**Figure 1:**
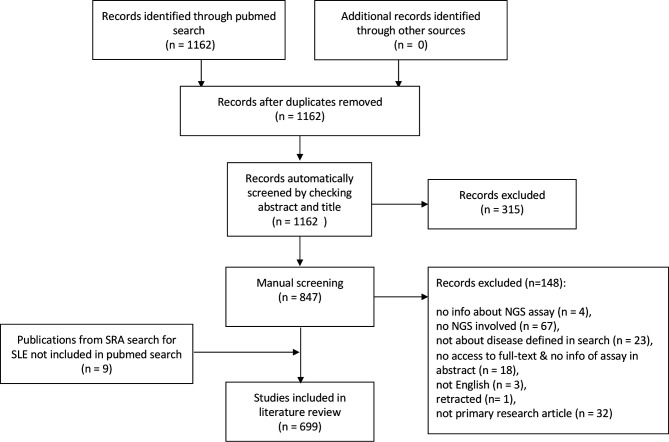
PRISMA flowdiagram of the literature review. For details, see the Methods section. NGS, next-generation sequencing; PRISMA, Preferred Reporting Items for Systematic Reviews and Meta-Analyses; SLE, systemic lupus erythematous; SRA, Sequence Read Archive.

This search was carried on 4 September 2020 and resulted in 1162 PubMed hits, which were (if possible) annotated regarding disease name, PubMed ID, assay, journal, year of publication by automatic screening the title and abstract. Reviews (ie, publications which have ‘Review’ in metadata) and commentaries were excluded and missing information was added manually by manual inspection of the publication. After manual curation, 699 studies were included in this literature review (figure 1).

A list of all identified publications can be found at https://github.com/sebboegel/pubmed_rheuma_HTS.

### SRA data analysis

Searching the SRA portal was carried out via the SRA portal at https://www.ncbi.nlm.nih.gov/sra using the diseases names identified in the literature review as key words one after another (ie, only one disease was searched at a time), then using the Run Selector (‘Send results to Run Selector’), switching to the old Run Selector (‘Revert to the old Run Selector’) and downloading the metatable, which was input to a custom-built python script extracting all necessary information. In addition, the python package *pysradb* 18 was used for retrieving PubMed identifiers for an associated SRA project number.

### Code availability

All scripts, input and result files, comments, as well all figures in this manuscript, generated with R package ggplot2 (V.3.2.1,^19^) are available at https://github.com/sebboegel/pubmed_rheuma_HTS.

### Paper counting

**Figure 2:**
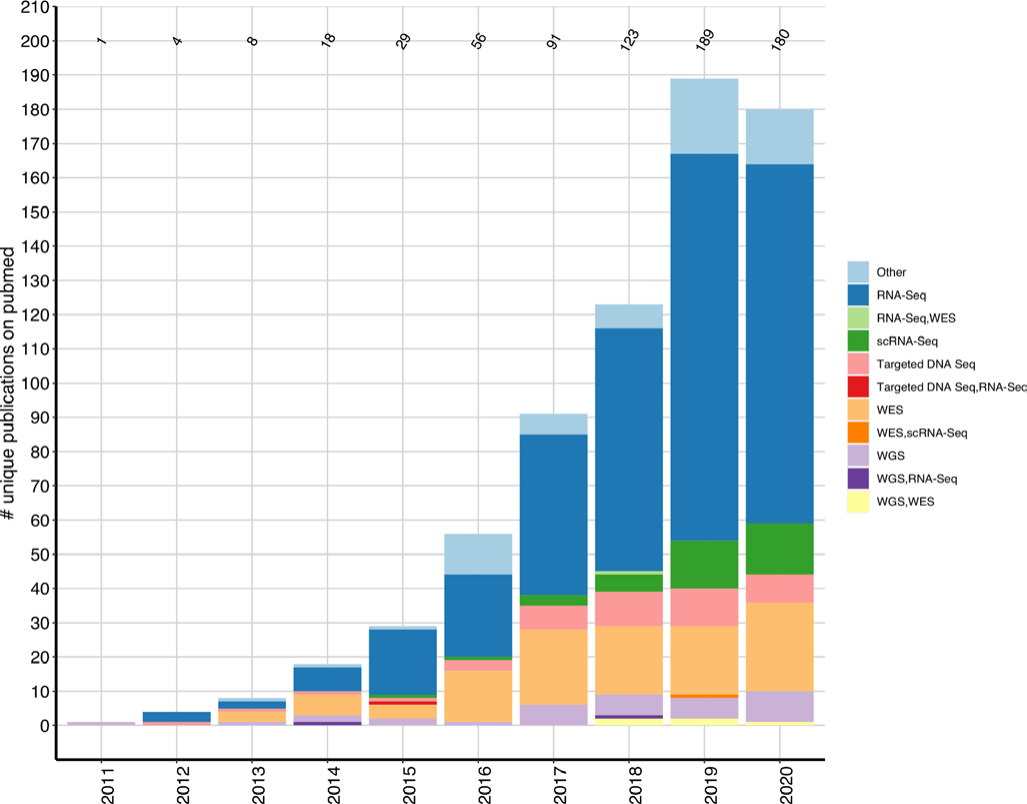
Publications per year. Number of unique identified primary research articles per year using different HTS assays in rheumatic diseases. HTS, high throughput sequencing; scRNA-Seq, single cell RNA-seq; WES, whole-exome sequencing; WGS, whole-genome sequencing.

**Figure 3:**
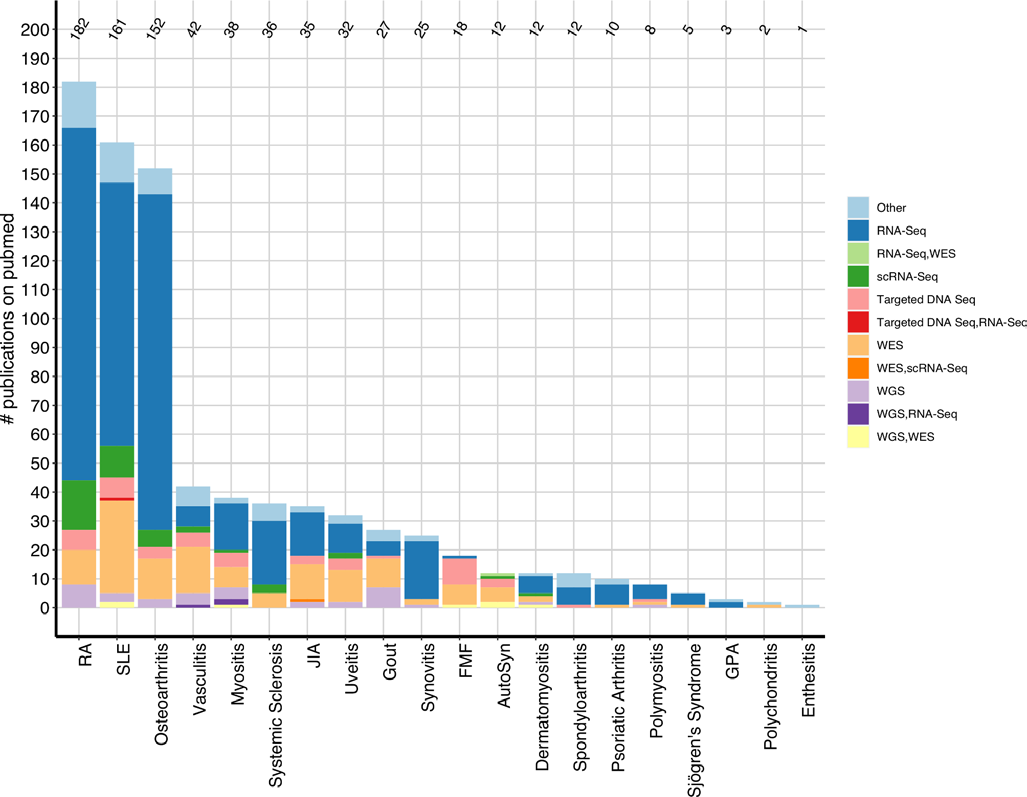
Publications and HTS assays per disease. Number of identified primary research publications per rheumatic disease using different HTS assays. AutoSyn, autoinflammatory syndrome; FMF, familial mediterranean fever; GPA, granulomatosis with polyangiitis; JIA, juvenile idiopathic arthritis; RA, rheumatoid arthritis; scRNA-Seq, single cell RNA-Seq; SLE, systemic lupus erythematosus; WES, whole-exome sequencing; WGS, whole-genome sequencing.

For counts that are not disease based (such as figure 2), the unique number of publications are depicted, which sum up to 699. However, as there exist publications using HTS on multiple rheumatic diseases, counting these papers in disease-based analysis (eg, figure 3) sum up to the total number of records (n=813), as a paper focusing on for example, SLE and RA will appear in the count for SLE and RA. Similarly, as there are publications using more than one HTS assay, summing up the number of assays discussed in the Results section will also exceed the number of unique publications.

## Results

The semiautomated search strategy, consisting of an R-script and manual curation, resulted in 699 unique PubMed hits (813 total records). We analysed the identified literature according to the year of publication, the rheumatic diseases, the different HTS assays used, the wide variety of applications and the journals, in which these studies appeared.

HTS assays are adapted in rheumatic research: the number of papers including HTS published has increased from 18 in 2014 to 123 in 2018 and 189 in 2019 (figure 2). As of 4 September 2020, already 180 studies have been published and following this exponential growth, up to ~340 studies can be assumed by the end of 2020 (online supplemental figure S1). One of the first HTS studies we identified with this search strategy was published in 2011 and used whole-genome sequencing (WGS) to identify low-frequency variants associated in gout.^20 21^

10.1136/rmdopen-2020-001324.supp1 Supplementary data

10.1136/rmdopen-2020-001324.supp2 Supplementary data

RA, n=182/699 unique publications, 26%, SLE, n=161, 23% and OA (n=152, 22%) are the rheumatic diseases with the most reported use of HTS assays (figure 3). Applications of HTS in these diseases range from HLA typing,^22^ TCR,^23 24^ BCR,^25 26^ and gene expression 27–29 profiling, as well as identification of T cell epitopes,^30^ antibody repertoires,^31^ and pathogenic mutations.^32 33^

The most represented assay is RNA-Seq (n=457, 65%) for the identification of biomarkers in blood or synovial tissue, for example, to distinguish active versus inactive/low disease activity states,^27^ to examine response to anti-TNF therapy in RA,^34^ to identify gene expression signatures correlating with disease phenotype,^35^ for longitudinal analysis of peripheral blood TCR diversity in patients with SLE,^36^ as well as for subgrouping patients with SLE with common clinical characteristics,^28^ characterisation of circulating memory stem T cells in RA,^37^ as well as to examine the BCR repertoire in patients with RA to identify B cell clones associated with autoreactivity.^38^ In addition to messenger RNA, a wide range of RNA types can be measured, such as microRNAs (miRNAs) in RA,^39^ JIA,^40^ SLE and Sjögren’s syndrome,^41^ long non coding RNA (lncRNA) in SLE^42^ as well as myositis,^43^ and finally circular RNA as biomarker in SLE^44^.

Transcriptomic analysis of individual cells (single cell RNA-Seq, scRNA-Seq) is increasingly becoming popular in cancer research,^45^ for example, to better capture tumour heterogeneity. Here, we identify 40 out of the 457 RNA-Seq studies (9%, online supplemental figure S2) uses scRNA-Seq with applications in, for example, SLE for mapping disease heterogeneity at the single-cell level using the blood transcriptome^46^ or for the identification of previously uncharacterised fibroblast subpopulations in the synovium of patients with RA.^47^

10.1136/rmdopen-2020-001324.supp3 Supplementary data

Applications for WES and targeted DNA (panel) sequencing (n=169, 24%) include identification of pathogenic mutations (mostly point mutations, small insertions and deletions) that can aid in diagnosis of monogenic autoinflammatory diseases and vasculitis,^48^ FMF,^49^ gout^50^ or familial RA, SLE and primary Sjögren’s syndrome^51^ or Uveitis.^52^ Of note, while HTS assays are powerful tools for large cohorts, we find many case reports using WES and gene panel sequencing in, for example, in a young patient with cutaneous vasculitis^53^ or JIA,^54^ as well as in a patient with RA experiencing immune dysregulation syndrome after abatacept therapy.^55^

We identified 42 (6%) studies using WGS. Again, the main application was identification of genetic variants, especially copy number variations, for example, of Fcγ receptor genes in RA^56^ and association of mitochondrial genetic variation and copy number with gout,^57^ as well as pharmacogenomic approaches examining patient’s response to golimumab treatment explained by common single-nucleotide variations.^58^

Other assays (online supplemental figure S3) include the analysis of bacterial species using HTS (metagenomics, n=33; 5%) in, for example, a joint infection in a patient with SLE,^59^ of the faecal microbiota of SLE mice^60^ or the lung microbiota in early RA,^61^ as well as epigenetic analysis (n=32, 5%) in SLE,^35 62^ RA,^63 64^ systemic sclerosis^65^ and finally, phage immunoprecipitation sequencing (n=1) for HTS of autoantibody repertoires in systemic sclerosis.^66^

10.1136/rmdopen-2020-001324.supp4 Supplementary data

Among the journals in which these studies appeared, ‘Arthritis and Rheumatology’ (n=58, 8%), ‘Annals of the Rheumatic Diseases’ (n=40, 6%) and ‘Plos One’ (n=29, 4%) are the leading journals publishing papers covering a broad range of HTS assays, whereas the journals ‘JCI Insight’ (n=12, 2%) and ‘Journal of Immunology’ (n=9, 1%) focused so far on RNA-Seq, and ‘Paediatric rheumatology online journal’ (n=9) focus on WES (online supplemental figure S4) for the identification of disease-relevant genetic variants.

10.1136/rmdopen-2020-001324.supp5 Supplementary data

### Raw-sequencing data in public domain

**Figure 4:**
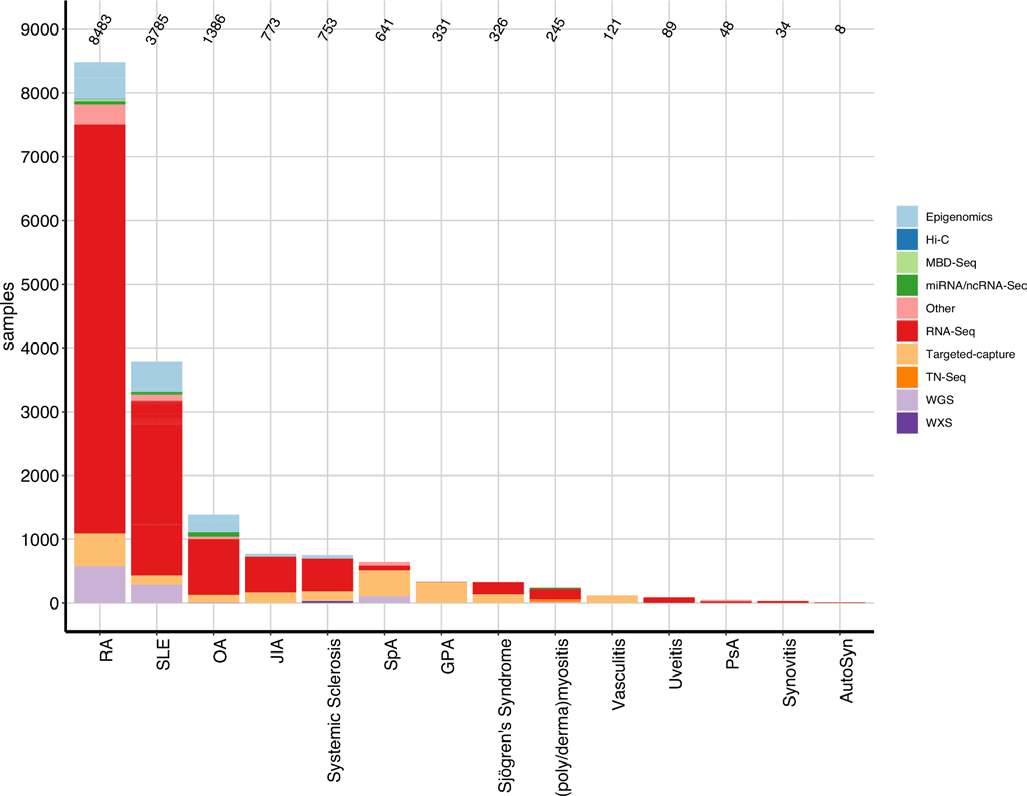
Public available high throughput sequencing (HTS) datasets. number of publicly available HTS samples on Sequence Read Archive for the rheumatic diseases identified in the literature review. AutoSyn, autoinflammatory syndrome; GPA, granulomatosis with polyangiitis; Hi-C, chromosome conformation capture; JIA, juvenile idiopathic arthritis; MBD-Seq, Methyl CpG binding domain-based capture and sequencing; miRNA-Seq, micro-RNA-Seq; ncRNA-Seq, non-coding-RNA-Seq; OA, osteoarthritis; PsA, psoriatic arthritis; RA, rheumatoid arthritis; SLE, systemic lupus erythematosus; SpA, Spondyloarthritis; TN-Seq, transposon insertion sequencing; WGS, whole-genome sequencing; WXS, whole-exome sequencing.

A search of samples in the SRA portal using the diseases identified in the PubMed search as key words revealed 17 023 HTS samples (figure 4) in 296 projects (online supplemental figure S5). The number of samples generated per study varies dramatically in the identified SRA projects (online supplemental figure S5) with 32/296 (11%) studies involving more than 100 study objects. Half of them (n=16) are produced in RA, seven in SLE, three in SpA, two in OA and JIA, and one in Systemic Sclerosis and GPA. The median number of HTS samples across the projects within the diseases is highest in GPA (72 samples/ study) and lowest in PsA 6.5 samples/ study). The vast majority of primary sequencing data originates from human biomaterial (15414/17023, 90.5%, online supplemental figure S6, primarily from samples reflecting the disease of interest (9854/17023, 58%, online supplemental figure S7, such as patients or disease models and 864 (5%) healthy controls. For the remaining 6305 (37%) samples, no phenotype or disease state was defined in the SRA metadata.

10.1136/rmdopen-2020-001324.supp6 Supplementary data

10.1136/rmdopen-2020-001324.supp7 Supplementary data

10.1136/rmdopen-2020-001324.supp8 Supplementary data

The majority of the samples are associated with RA (n=8483, 50%), SLE (n=3785, 22%) and OA (n=1386, 8%) and correlate with the relative abundance of studies identified in the literature search for these diseases. Also, the dominance of the RNA-Seq assay is consistent with the PubMed findings. However, there are obvious inconsistencies when comparing the number of publications using or producing HTS data (figure 3) with the number of projects depositing HTS data on SRA (online supplemental figure S5). To examine this discrepancy, we used the RNA-Seq assay (including scRNA-Seq, miRNA, ncRNA) in SLE as an example for in depth analysis. By using the metadata table on the SRA website and a customised python script, followed by manual inspection, we identified 56 SRA projects, of which 43 projects provide raw RNA-Seq data. For seven of them no corresponding publication could be identified. Of the remaining 36 Projects, two SRP-IDs are associated with the same publication and two SRP-IDs are each associated with two different publications, resulting in 37 PubMed-IDs associated to SRA-Projects, which overlap with the 107 RNA-Seq studies in SLE identified in the PubMed search (figure 3). The remaining 70 publications were examined manually and 32/70 publications provided no information on the availability of the raw sequencing data at all, 13/70 provide the raw data ‘on reasonable request’, nine studies did not produce RNA-Seq data, but rather used publicly available datasets, six papers could not be accessed, three studies deposited the raw data at the European Genome Archive (EGA), two publications report an embargo on the data, that is, it will be provided with delay after the acceptance of the manuscript and one study made the data available under protected access at the database of Genotypes and Phenotypes (dbGaP) (online supplemental figure S8). Of note, four studies not providing the raw sequencing are case reports, which is consistent with FMF consisting primarily of case reports and we do not find any sequencing data from FMF on SRA (figure 4).

10.1136/rmdopen-2020-001324.supp9 Supplementary data

The most prominent sequencing platform is the Illumina HiSeq series (n=13 063, 77%, online supplemental figure S9) and paired end as preferred read layout (n=11 533, 68%), except for SLE with 1300 paired end and 2485 single end reads samples (online supplemental figure S10).

10.1136/rmdopen-2020-001324.supp10 Supplementary data

10.1136/rmdopen-2020-001324.supp11 Supplementary data

**Figure 5:**
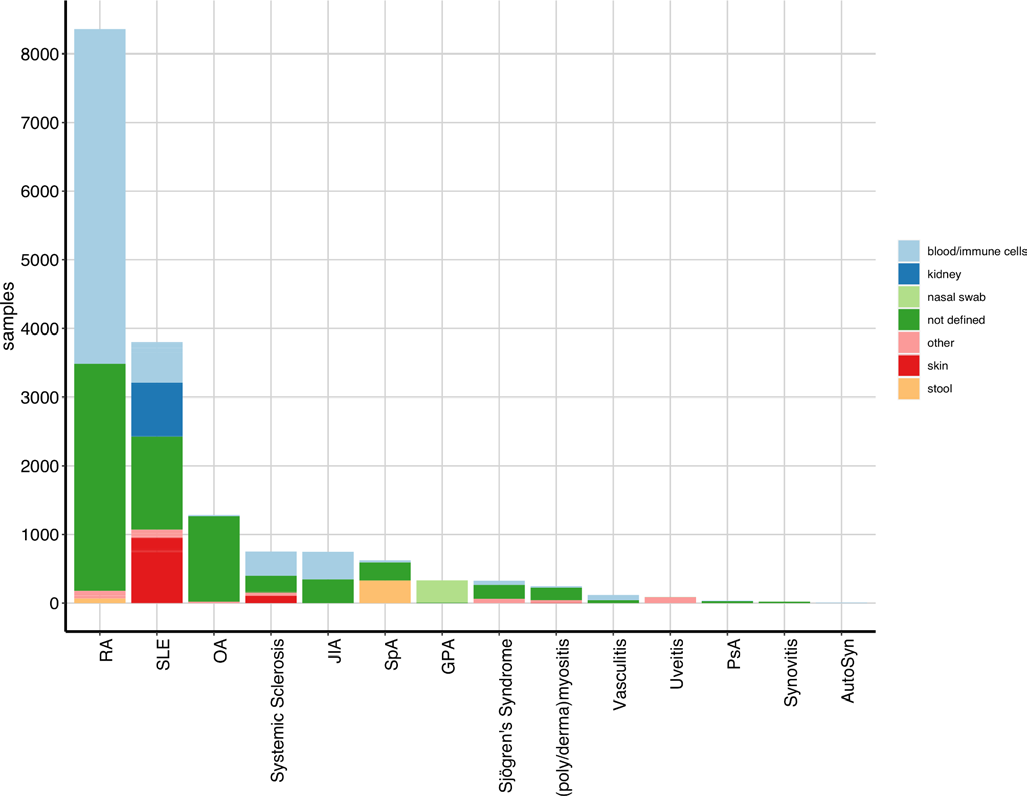
Tissue source of high throughput sequencing data on Sequence Read Archive (SRA). Distribution of tissues subject to sequencing in publicly available datasets on SRA. Disease abbreviations as in figure 4.

Analysing the tissue source of the HTS sample across different diseases (figure 5) reveals blood (whole blood, plasma, serum, peripheral blood mononuclear cell) and isolated immune cells (T cells, monocytes, dendritic cells) as the primary source material (6461/17 023, 38%). There are disease-specific preferences such as, cartilage in OA (84% of samples with defined tissue source), stool (faeces) in SpA, 87%, kidney in SLE (33%), synovium in PsA (52%) and synovitis (100%), as well as salivary gland in Sjögren’s syndrome (50%), muscle in (poly/derma)myositis (63%) and retina in uveitis (100%) (figure 5 and online supplemental figure S11, ‘sra_tissue.tsv’).

10.1136/rmdopen-2020-001324.supp12 Supplementary data

### Reporting of clinical patient data

Next, we examined the availability and quality of clinical information about the patients that were subject to sequencing and which HTS data is available from SRA. There are two challenges in finding patient characterisation of the primary HTS data of interest. First, the associated metadata does not use a defined ontology and no standardised patient/sample characterisation is required when deopsiting the sequencing data on SRA. Second challenge is the identification of the publication associated with the data. If no PubMed identifier is provided in the respective bioproject on SRA, the study can occasionally be identified by searching the bioproject title on PubMed or a related search engine.

In general, reporting of clinical data was highly diverse. In order to quantify this diversity, we used the RNA-Seq assay (including scRNA-Seq, miRNA, ncRNA) in SLE as an example for a detailed analysis. Of the 43 SRA projects providing SLE RNA-Seq data, 23 contain sequencing data from SLE patients, whereas the remaining projects deal with model organisms and cell lines (n=12) or the associated publications could be neither found (n=7) or accessed (n=1). Of these 23 projects, three associated manuscripts contain no information about the sequenced patient, four studies have at least a rudimentary set of information, eight publications with a medium set and eight papers with very detailed reporting of patient characteristics (table 1 and online supplemental figure S12).

10.1136/rmdopen-2020-001324.supp13 Supplementary data

**Table 1:** Examples of reporting clinical data for SLE patients subject to RNA-Seq

Category	Information provided	Bioproject	SRP-ID	Reference
No information	–	PRJNA431313	SRP131173	102
Rudimentary set	Demographics (age, sex, race)	PRJNA505280	SRP168421	103
Medium set of useful information	Demographics, serology, medication	PRJNA514365	SRP178271	104
	Demographics, disease duration, SLEDAI	PRJNA379992	SRP103040	105
Advanced set of useful information	Demographics, disease duration (years from diagnosis), SLEDAI, medication at time of blood draw (mg/day), ANA reactivity, prominent clinical features (such flares, new rash, etc), information for each patient individually.	PRJNA484966	SRP156584	106
	Demographics, SLEDAI, severity, immunosupprevise therapy, disease manifestation, comorbidities, medication, serology (eg, anti-DNA, C3/4)	PRJEB24742	SRP189104	107

Full list of patient characteristics available in file ‘SLE_pubmed_rna_patient_info.csv’ (see data availabiliy).

Bioproject: accessible at https://www.ncbi.nlm.nih.gov/bioproject/.

ANA, anti-nuclear antibody; C3/4, complement 3/4; SLE, systemic lupus erythematous; SLEDAI, Systemic Lupus Erythematosus Disease Activity Index.

## Discussion

High throughput gene expression profiling using DNA microarrays have already provided unprecedented views into the blood transcriptome of, for example, SLE,^67 68^ RA,^69^ SpA,^70^ and thus paved the way for the development of personalised diagnostic and therapeutic strategies.

The introduction of ‘next generation’ HTS platforms, together with a tremendous evolution of open source bioinformatic software, enables the rapid detection of a wide variety of molecular features, such as alternative splicing events, RNA editing, HLA typing, BCR and TCR typing, mutation detection and many more,^5^ thus adding new dimensions in understanding disease pathogenesis and biomarker identification.^71^ Application and impact of HTS using NGS platforms in rheumatology have been reviewed in general^12^ and for individual diseases, such as SLE^72^ or RA.^73^

However, this is to our knowledge, the first study quantifying the usage of HTS in rheumatological research by reviewing literature on PubMed and examining public HTS data on SRA.

A limitation of this approach is that the numbers identified in this search are likely to be underestimated as potential publications may have been missed by the search. For example, one of the first studies using HTS for TCR and BCR repertoire analysis in RA was published April 2011^74^ and is not indexed on PubMed (and thus has not been found by this search). Further, there exist more than 200 different rheumatic diseases^75^ and our approach identified only a small subset (n=20). The strength of this approach is that it is easily reproducible. The provided R and python scripts along with all input and result files as well as comments about the manual steps of the analysis, enable reproduction of the results presented here and can be adopted for allowing literature review at any time point in the future.

A key finding is that HTS is indeed being adapted in rheumatological research with an exponential growth rate in number of publications since 2011. Major indications are RA and SLE, which are rheumatic disease with high prevalence rates of 0.5%–1% of the adult population in RA and 20–150 SLE cases per 100 000 individuals in the USA^76 77^ in contrast to the many other rheumatic conditions that are classified as ‘rare disease’, such polymyositis (prevalence 1/14 000^78^). For the majority of the indications identified in this review, RNA-Seq was the most represented assay. While analysis of nucleotide variations by exome and genome sequencing holds great promise in the diagnosis of rare diseases,^79^ going beyond the exome/genome, for example, analysing the gene expression to learn about pathomechanisms or personalised medicine approaches^12^ results in the major challenge of very small patient populations.^80^ Indeed, we find that the majority of studies depositing sequencing data on the public repository SRA included low numbers of samples posing a challenge to the application of classical statistical analyses for target identification.^81^ However, to be fair, not all projects we identified were designed to find biomarkers, such as case reports or mechanistic experiments using cell lines or model organisms.

The second key finding is that there exists a large number of raw sequencing data on the public repository SRA. However, we identified a gap between publications reporting usage of HTS assays and availability of this data on SRA. We quantified this gap with RNA-Seq projects for SLE as an example and found that the majority of studies not depositing data on SRA, do not provide any information about the availability of the primary sequencing data in the publication. Second most common finding was the information on the availability ‘on request’. Reasons that might hinder researchers making HTS data publicly available might be technical or privacy challenges in sharing genomic data^82 83^ or interests of the data owners.^84^ With regard to privacy concerns, a feasible solution could be the deposition in repositories providing controlled and protected access to genomic data, such as the ‘European Genome-Phenome Archive’ (EGA)^85^ or the ‘database of Genoytpes and Phenotypes’ (dbGaP).^86^ EGA stores genomic data of 2953 studies^87^ of which 1315 (45%) belong to ‘cancer’ and only 85 (3%) are labelled as ‘Inflammatory’ containing RA (n=19, 0.6%), SLE (n=7, 0.2%), ankylosing spondylitis (n=7) and psoriasis (n=1, 0.03%) datasets. As an example, very recently Panousis *et al* published a comprehensive genetic and transcriptomic profiling of 142 patients with SLE and 58 controls^27^ and provided the raw and processed HTS data, clinical phenotypes/covariates, as well as the results of the genetic analysis under protected access (one needs to apply to access this data) at https://ega-archive.org/studies/EGAS00001003662. dbGaP is an online repository created by the National Center for Biotechnology Information provides controlled access to large-scale genomic datasets with associated phenotypes, such as ‘The Cancer Genome Atlas’ (TCGA)^88^ or ‘Genotype-Tissue Expression’.^89^

Sharing HTS data have several advantages. First of all, when data are made available for reuse, citations to the initial report increase.^90^ In addition, genomic data potentially has value beyond the initial purpose and re-analysis of publicly available sequencing data with novel bioinformatic tools can lead to novel insights, for example, in RA,^91^ to examine HLA and proteasome expression in different tissues^92^ or public HTS data can be used to provide supportive information in addition to own sequencing experiments, as in the case of uncovering distinct subsets of patients with SLE using machine learning methods.^93^ However, clinically useful and translational reanalysis requires (1) the searchability of this data, which is only guaranteed if the data are deposited one of the above-mentioned repositories and (2) the availability of detailed patient characteristics along with clinical information linked to the respective sequencing sample (ie, data characterisation challenge).^94^

Very recently, Gossec *et al* present 10 EULAR points to consider (PTC) for the use of big data, including ‘omics and imaging data, in rheumatic and musculoskeletal diseases.^95^ Here, we emphasise the importance of clinical data linked to the patient HTS data and propose an additional PTC: ‘provide clinical characterisation’. It is necessary to agree on a set of rules for reporting clinical data in the context of genomic sequencing experiments, link them to the respective sequencing sample of the patient to connect genotype (eg, genome) with phenotype (eg, treatment response, organ manifestation, grade of disease) and extract as much clinically translatable information as possible from existing data. A successful example from cancer research is TCGA, which is a cancer genomics programme consisting of research centres worldwide, generating genomic, epigenomic, transcriptomic and proteomic data of more than 30 cancer types including histopathological images and clinical data. To make clinically valuable analysis comparable between the projects within the consortium, such as survival outcome analysis^96^ guidelines on reporting clinical data were developed^97^ and a data dictionary was defined to define necessary clinical entities, such as ‘Demographic’, ‘Diagnosis’, ‘Family History’, ‘Treatment’ and ‘Follow-up’.^98^ We recognise that there is rheumatic disease specific information that is important to share, for example, Schirmer test for Sjögren syndrome. Nevertheless, we translate these guidelines into the world of rheumatology and propose a minimal set of clinical data to be reported in HTS experiments (table 2).

**Table 2:** Proposal of a minimal set of clinical information when sharing patient HTS data to enable clinically useful reanalysis

Clinical entity	Data points
Demographic	ethnicity, gender, age
Diagnosis	Primary diagnosis (ICD-10), type/grade/stage, disease activity scores (SLEDAI, BASFI, BASDAI, VAS, DAS, …)
Exposure	cigarettes_per_day, years smoked
Family history	History of autoimmune disease in family
Follow-up	BMI, comorbidities, progression or recurrence, weight, disease duration
Molecular tests	Anti-CCP, HLA status, C3, C4, autoantibodies (ANA, ENA…)
Treatment	Therapeutic agents, dose, frequency, outcome, adverse events

ANA, anti-nuclear antibody; BASDAI, Bath Ankylosing Spondylitis Disease Activity Index; BASFI, Bath Ankylosing Spondylitis Functional Index; C3/4, Complement 3/4; CCP, Citrullinated Peptide/Protein antibodies; DAS, Diseases Activity Score; ENA, Extractable Nuclear Antigen; HLA, Human Leukocyte Antigen; HTS, high throughput sequencing; ICD, International Statistical Classification of Diseases and Related Health Problems; SLEDAI, Systemic Lupus Erythematosus Disease Activity Index; VAS, Visual Analog Scale.

Being already established in cancer research and in the field of Mendelian diseases,^99^ rheumatic diseases are about to become the third disease domain for HTS. This is an important observation, as many of the bioinformatic tools for analysing HTS data have been developed in the context of cancer research. Not all of them can be directly applied to rheumatology, such as mutation detection tools, and require adoption to rheumatological datasets. We foresee an evolution of bioinformatic software newly developed or adopted to the specific needs and questions of rheumatological disease. Especially the RNA-Seq assay, which we found already widely adopted in rheumatology, will be a central and powerful assay in deciphering pathomechanisms, precision approaches and might lead to new disease definitions based on molecular characteristics as it has been shown in cancer.^100^ However, there is a need for a global solution for sharing clinical and genomic data.^101^ This discussion started in cancer research and must continue in rheumatic research.
