# Correction: Current status of use of high throughput nucleotide sequencing in rheumatology

**DOI:** 10.1136/rmdopen-2020-001324corr1

**Published:** 2021-01-12

Boegel S, Castle JC, Schwarting A. Current status of use of high throughput nucleotide sequencing in rheumatology. *RMD Open* 2021;7:e001324. doi: 10.1136/rmdopen-2020-001324

The article has been corrected since it was published online. The funding statement has been updated as follows.

We wish to acknowledge the RARENET EU-Interreg for supporting this study. Furthermore, SB wishes to acknowledge and thank the Mainz Research School of Translational Biomedicine (TransMed) for support.

