# Supplementary material for: Current status of use of high throughput nucleotide sequencing in rheumatology
Source: RMD Open. 2021 Jan 6;7(1):e001324. doi: 10.1136/rmdopen-2020-001324 (PMC7789458; doi:10.1136/rmdopen-2020-001324)
Supplement: Supplementary data [file rmdopen-2020-001324supp001.pdf]

**Supplemental figure legends**

**Figure S1. Publication per year** with fitted exponential curve.

Figure S2. Single-cell RNA-Seq publication per year.

**Figure S3. Publications per disease II.** Number of identified primary research publications per rheumatic disease using different HTS assays labeled as "Other" in Figure 3.

**Figure S4. Publications per journal.** Number of identified primary research publications per journal using different HTS assays in rheumatic disease. Assay abbreviations as in Figure 3.

**Figure S5. Samples per SRA project.** Each point represents a SRA project depositing HTS data from a certain number of samples. On the upper side, the total number of projects for each disease is depicted. The median number of samples across all projects within a disease is depicted inside the boxplot. Disease abbreviations as in Figure 4.

**Figure S6. Species information for HTS data on SRA.** Disease abbreviations as in Figure 4.

**Figure S7. Phenotypic information for HTS data on SRA.** Disease abbreviations as in Figure 4.

**Figure S8.** Information about raw sequencing data availability in publication.

**Figure S9. Sequencing platform information for HTS data on SRA.** Disease abbreviations as in Figure 4.

**Figure S10. Read layout information for HTS data on SRA.** Disease abbreviations as in Figure 4.

**Figure S11. Tissue source of HTS data on SRA II.** Distribution of tissues subject to sequencing in publicly available datasets on SRA labeled as "Other" in Figure 5. Disease abbreviations as in Figure 5.

**Figure S12.** Availability of patient characterization for SLE RNA-Seq data.
