# Supplementary figures and images for: Current status of use of high throughput nucleotide sequencing in rheumatology
Source: RMD Open. 2021 Jan 6;7(1):e001324. doi: 10.1136/rmdopen-2020-001324 (PMC7789458; doi:10.1136/rmdopen-2020-001324)

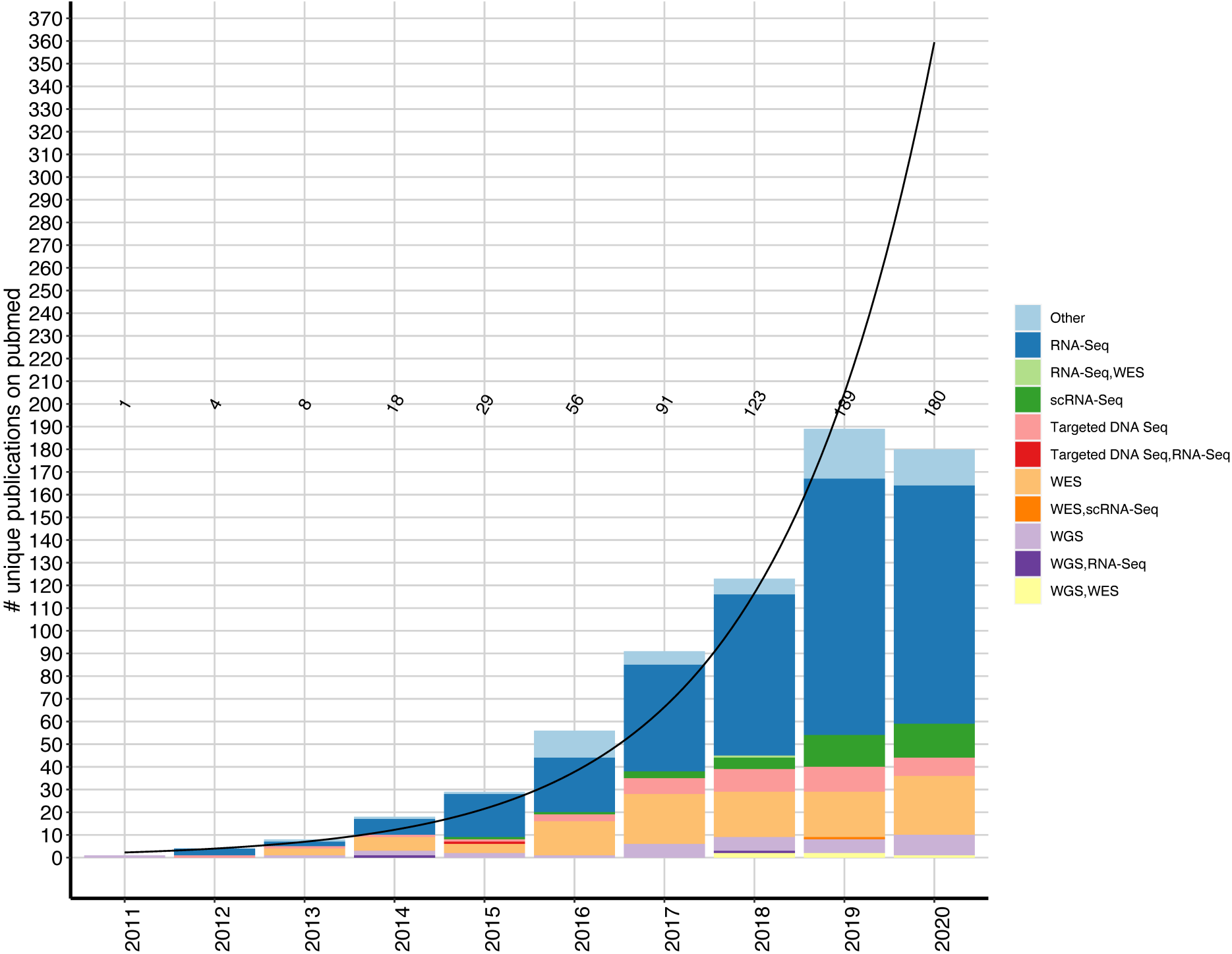

Supplement: Supplementary data [file rmdopen-2020-001324supp002.pdf]

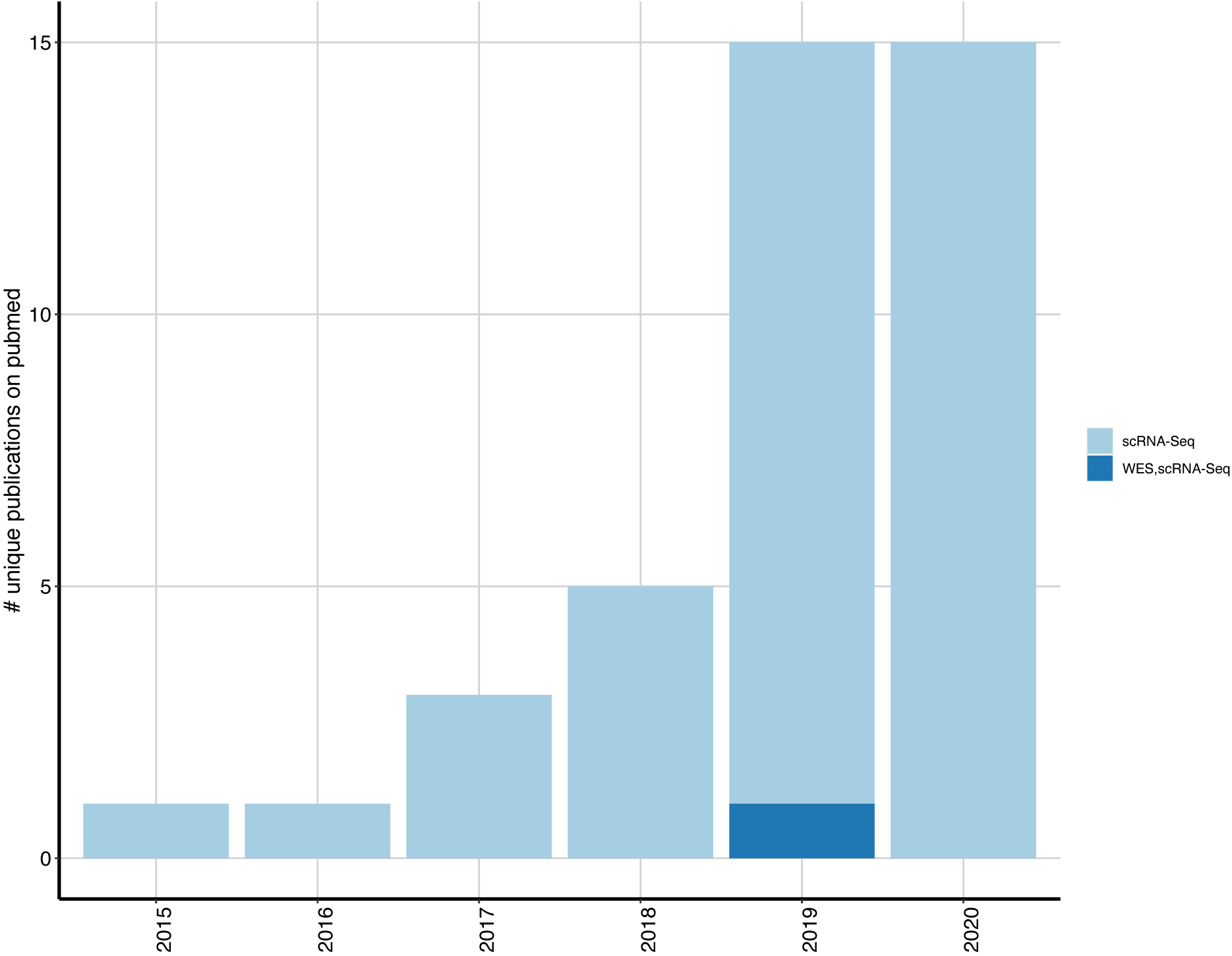

Supplement: Supplementary data [file rmdopen-2020-001324supp003.pdf]

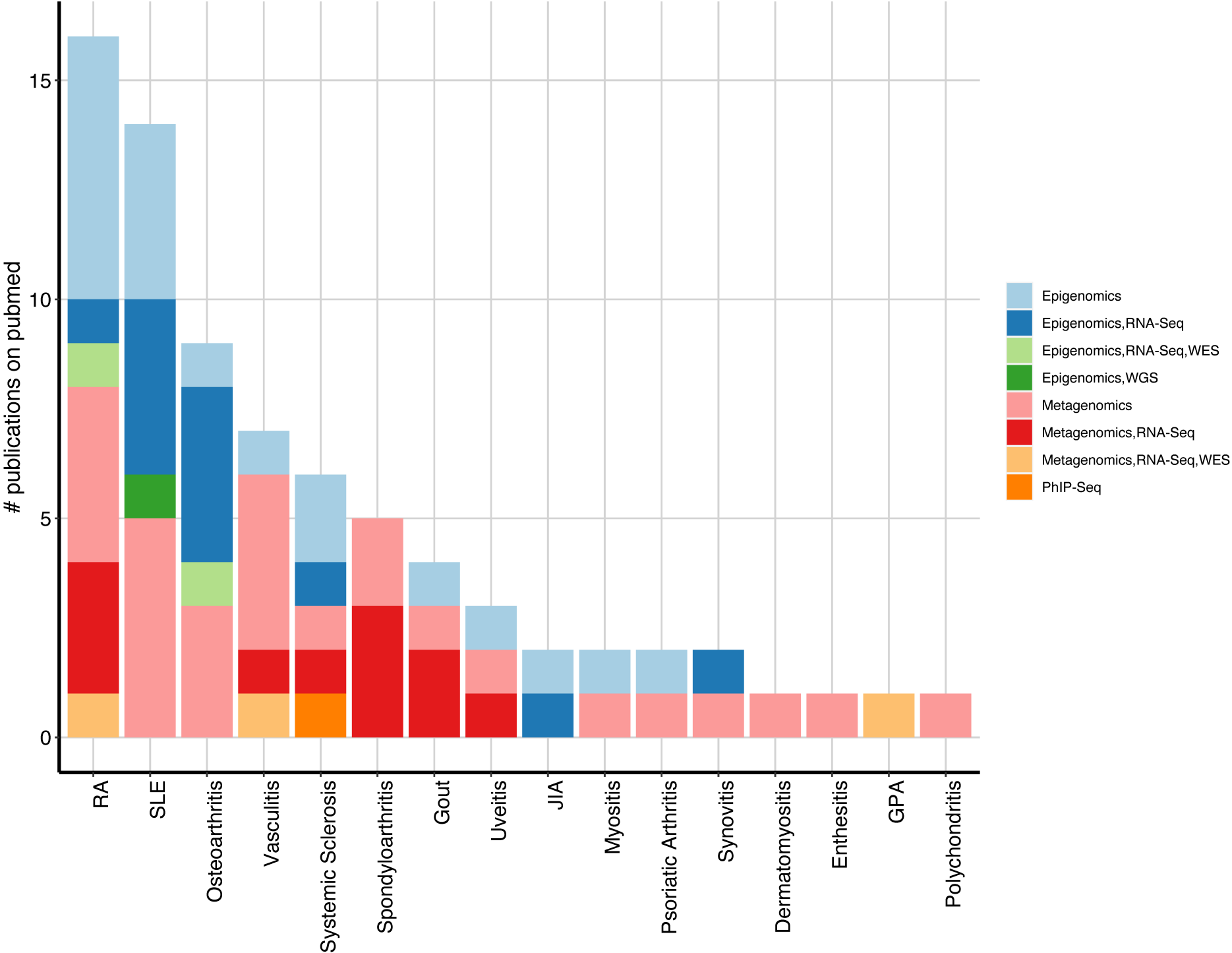

Supplement: Supplementary data [file rmdopen-2020-001324supp004.pdf]

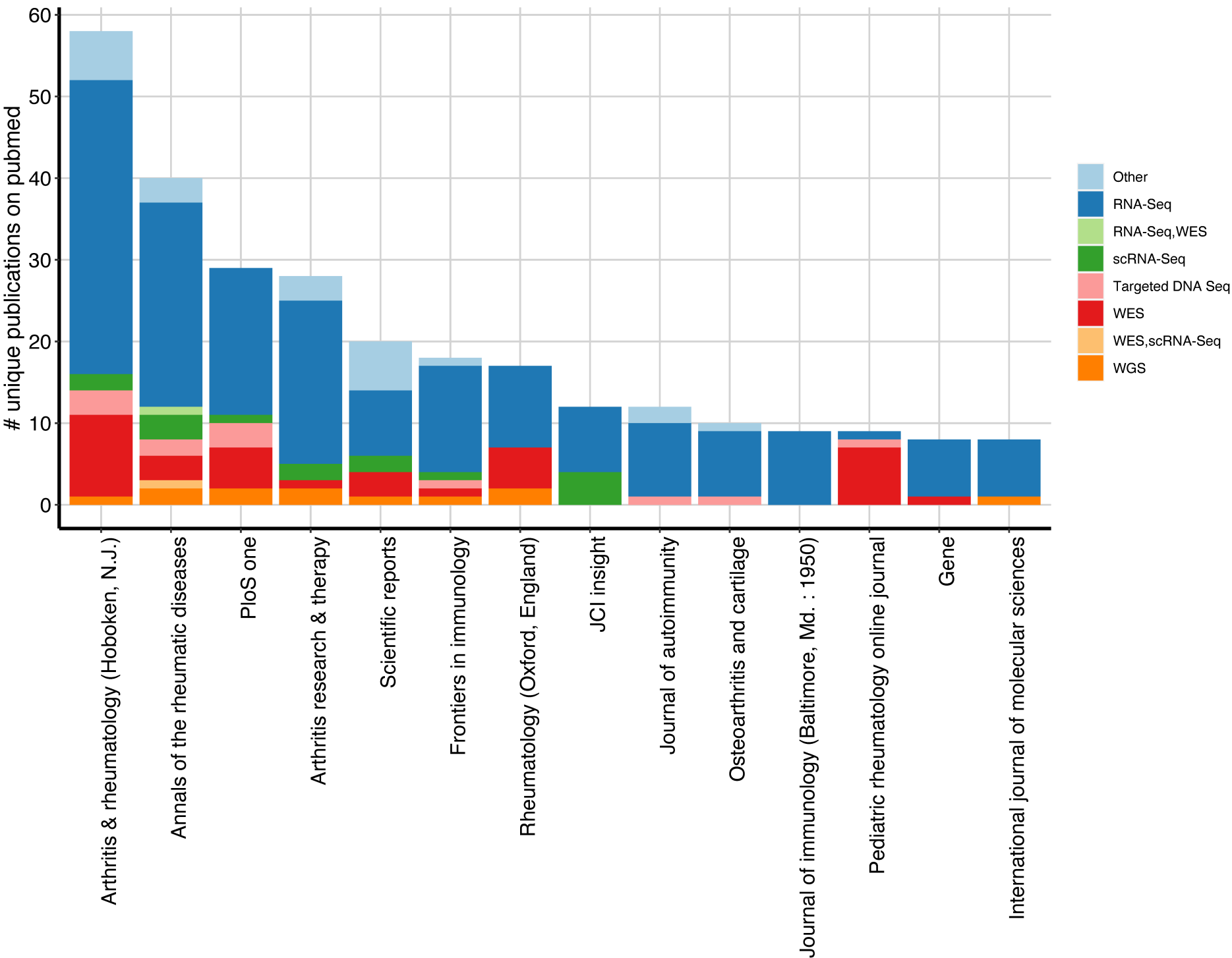

Supplement: Supplementary data [file rmdopen-2020-001324supp005.pdf]

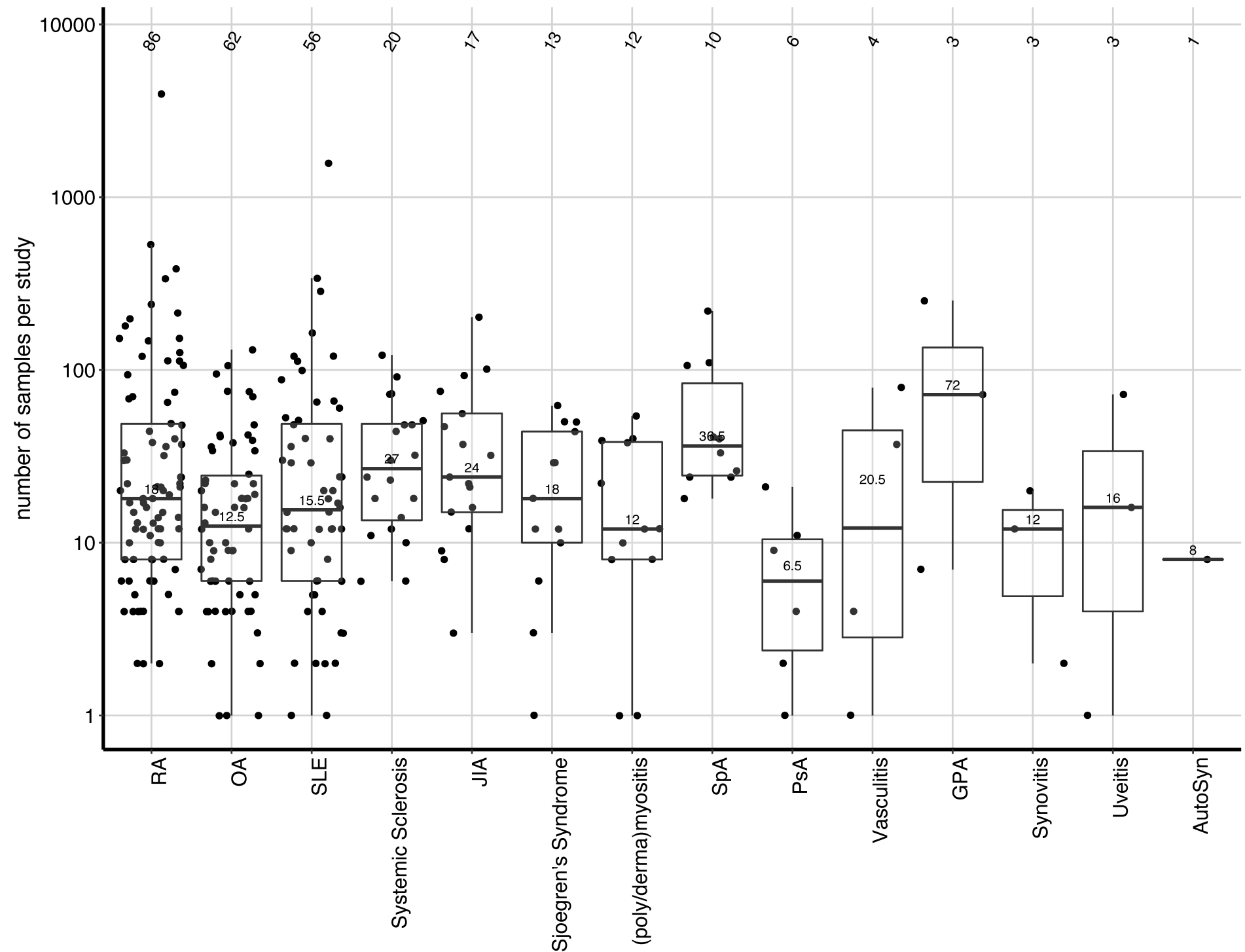

Supplement: Supplementary data [file rmdopen-2020-001324supp006.pdf]

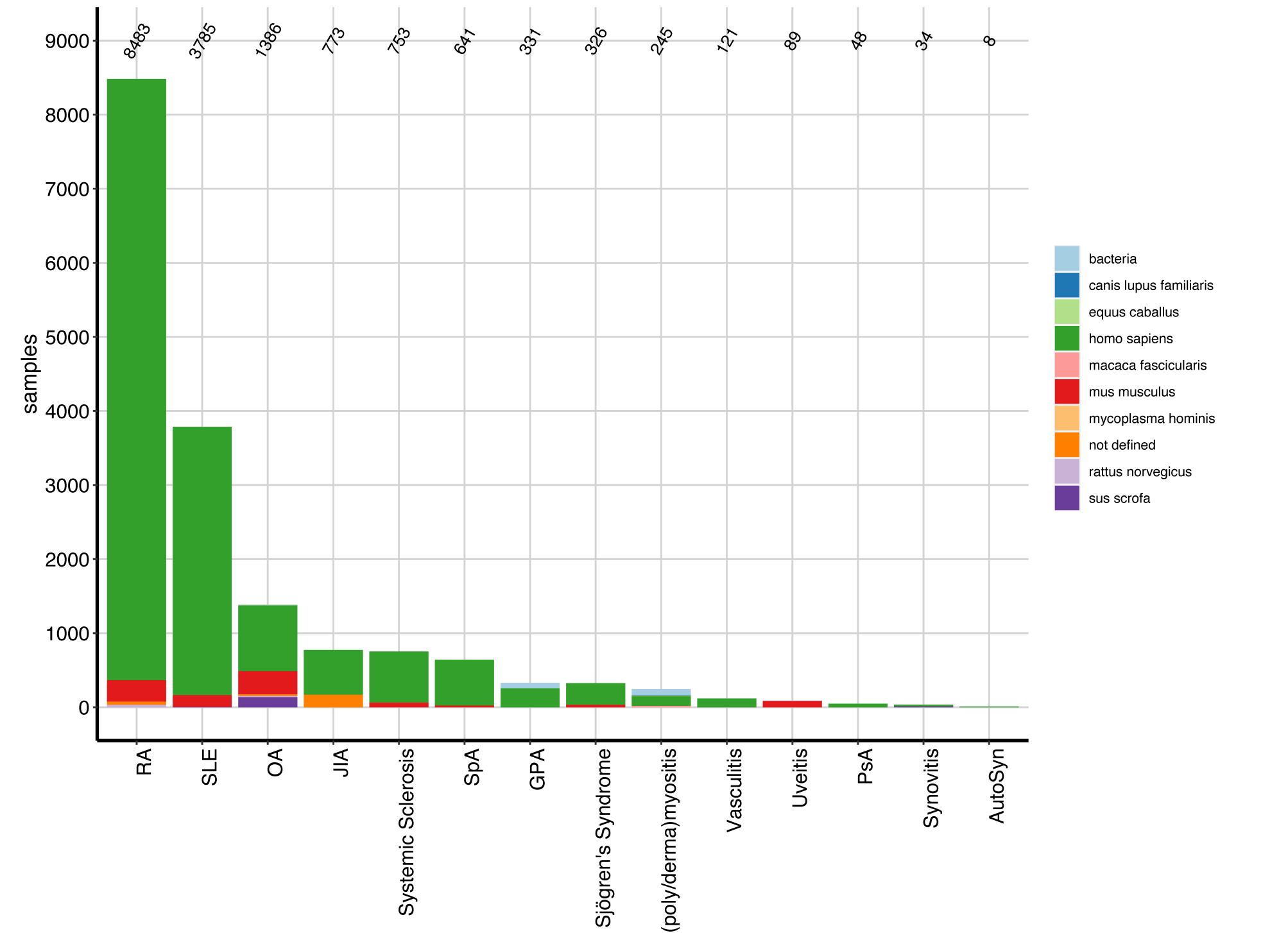

Supplement: Supplementary data [file rmdopen-2020-001324supp007.pdf]

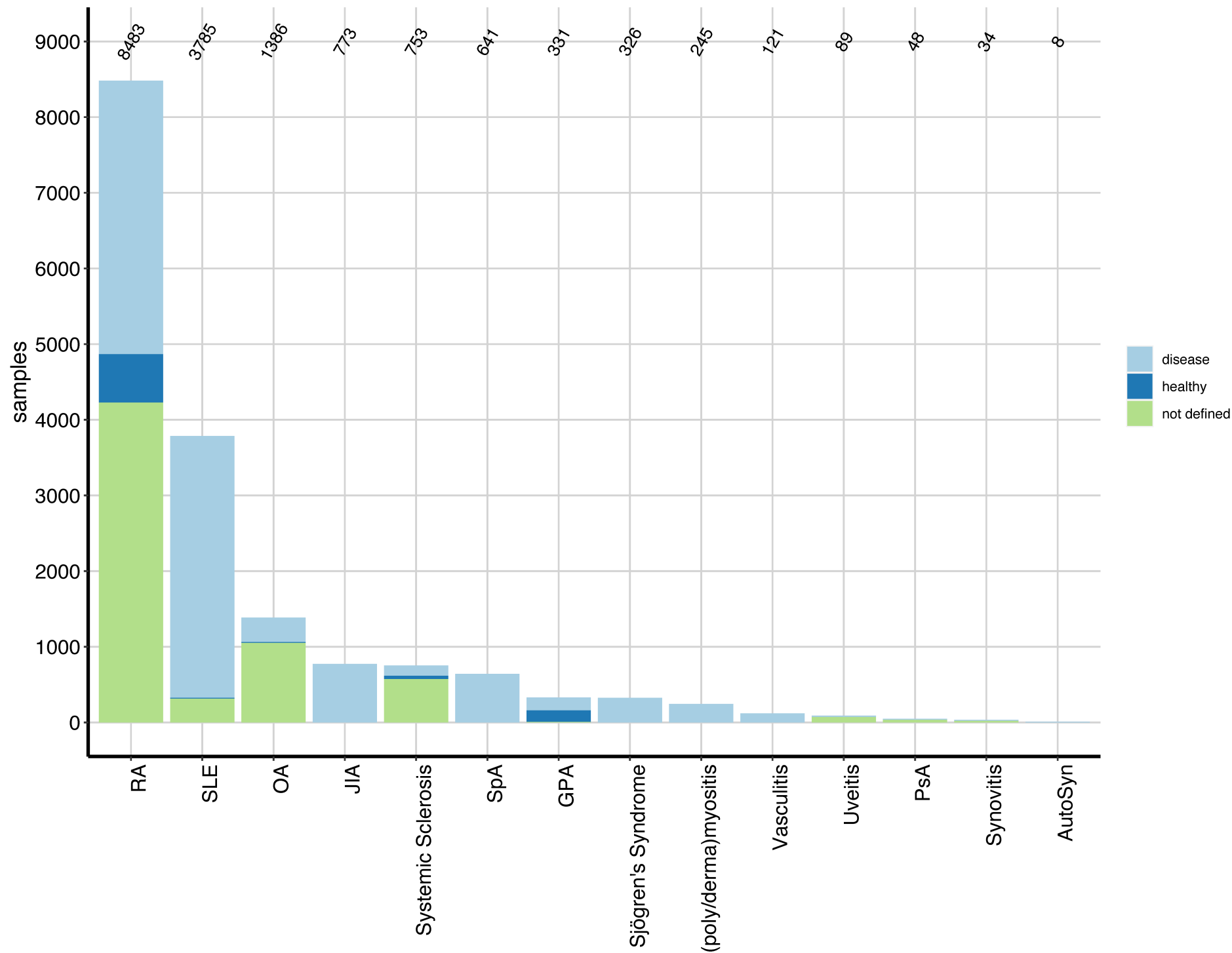

Supplement: Supplementary data [file rmdopen-2020-001324supp008.pdf]

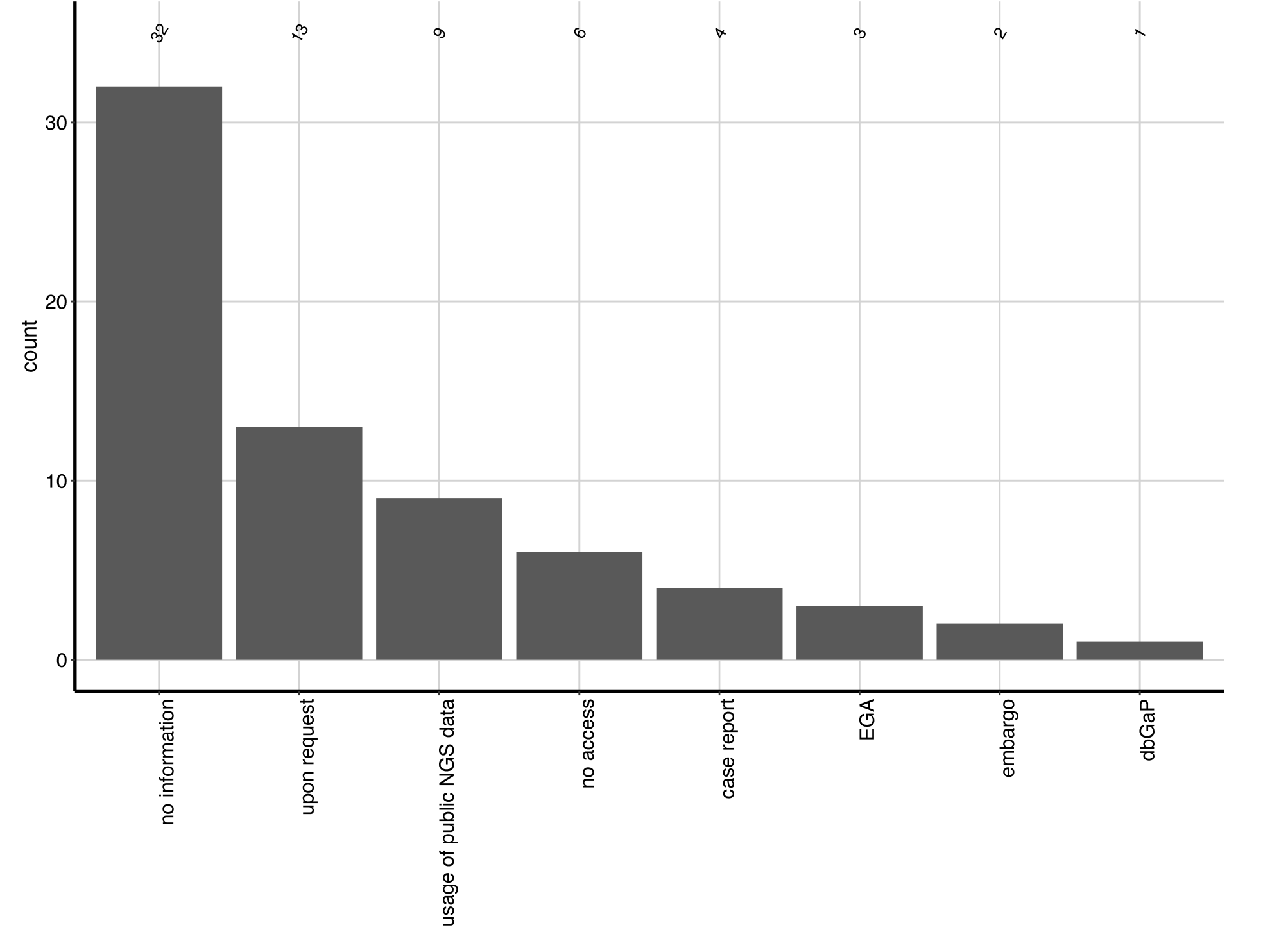

Supplement: Supplementary data [file rmdopen-2020-001324supp009.pdf]

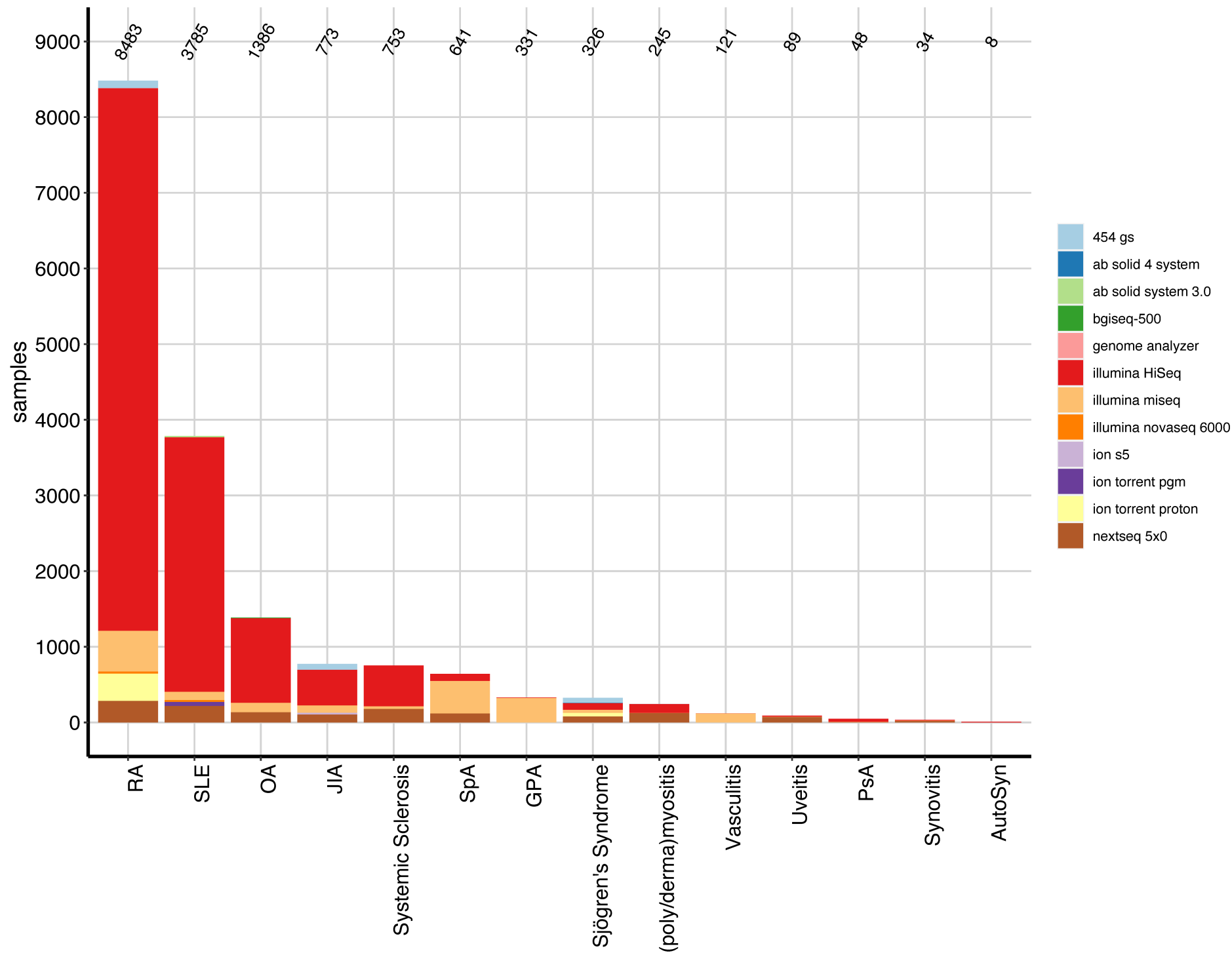

Supplement: Supplementary data [file rmdopen-2020-001324supp010.pdf]

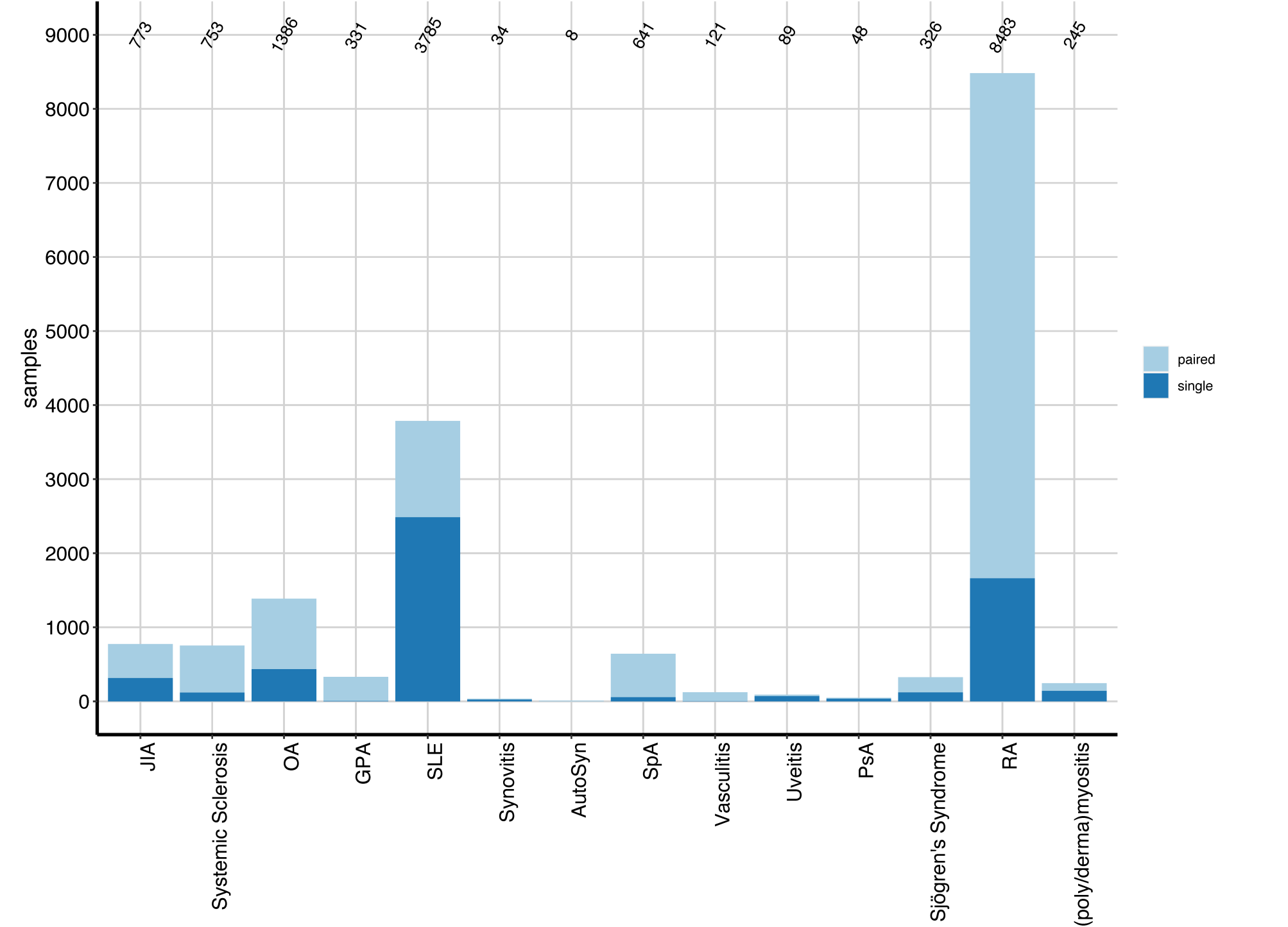

Supplement: Supplementary data [file rmdopen-2020-001324supp011.pdf]

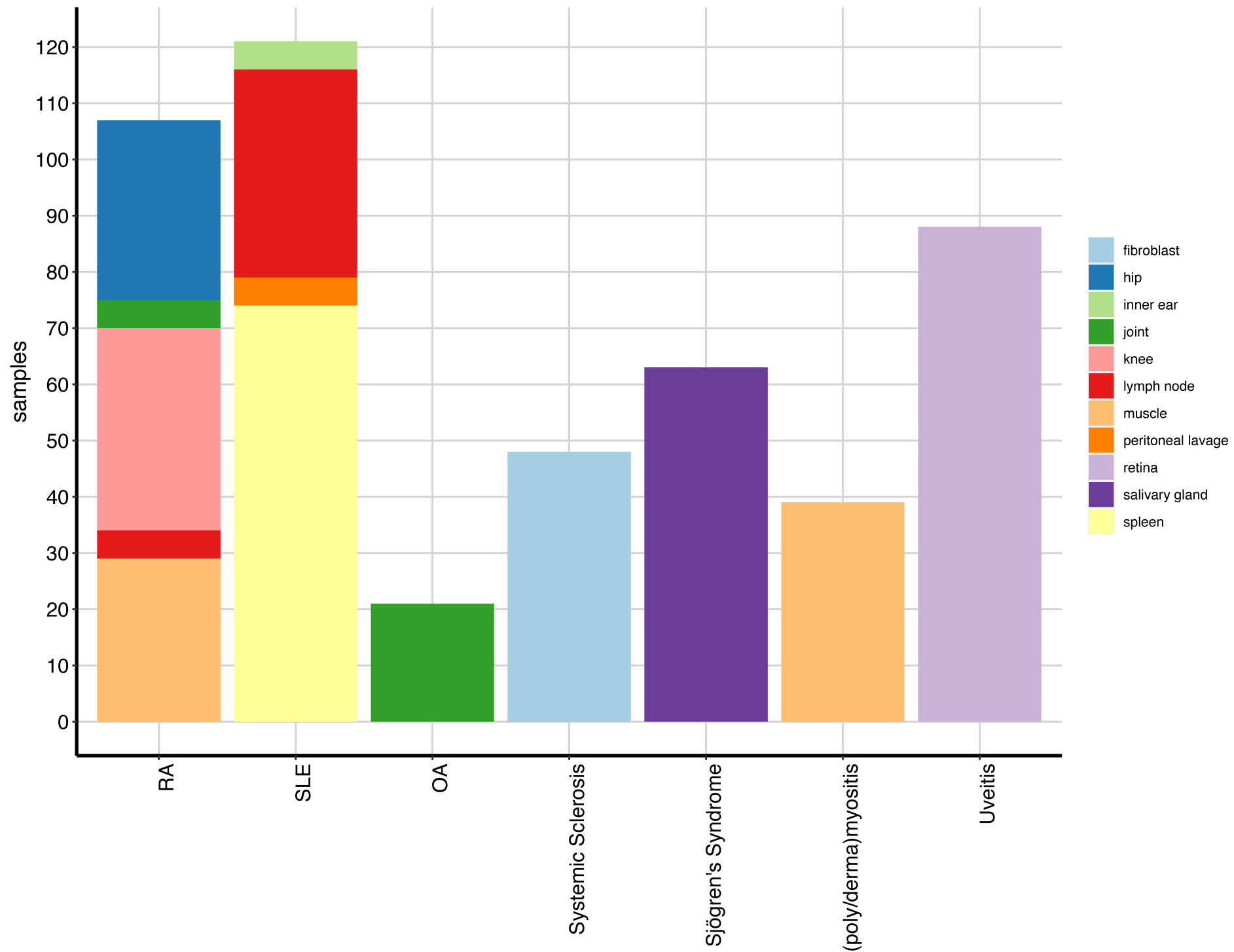

Supplement: Supplementary data [file rmdopen-2020-001324supp012.pdf]

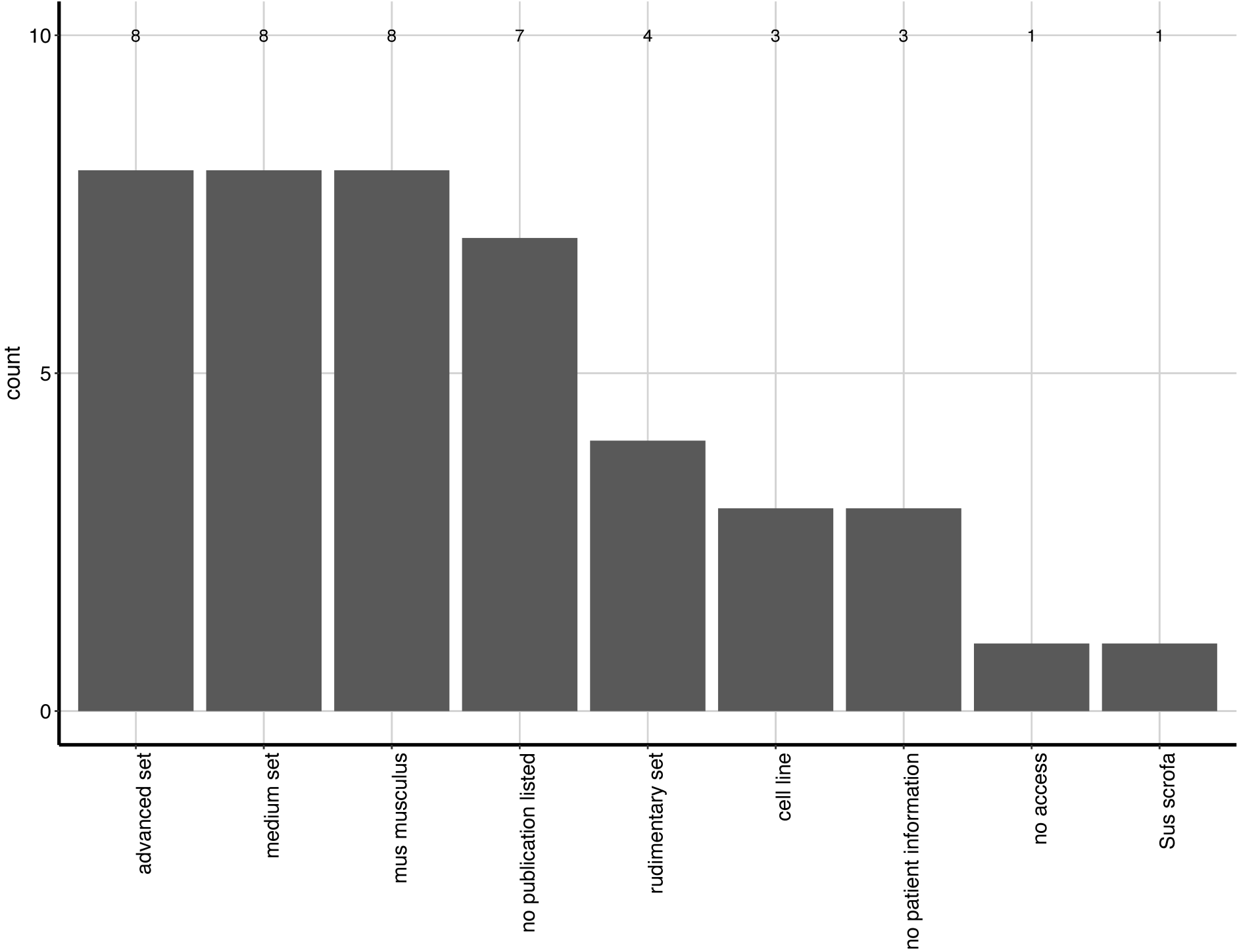

Supplement: Supplementary data [file rmdopen-2020-001324supp013.pdf]
